# Curcumin enhances the effects of TRAIL in TRAIL-resistant non-small cell lung cancer cell lines

**DOI:** 10.1186/s12906-026-05398-z

**Published:** 2026-05-06

**Authors:** Nurul Izzah Mohd Pauzi, Noor Atiqah Fakharuzi, Nor Rizan Kamaluddin, Moon Nian Lim, Yuslina Mat Yusoff, Adiratna Mat Ripen, Kamal Shaik Fakiruddin

**Affiliations:** 1 Haematology Unit, Cancer Research Centre, Institute for Medical Research (IMR), National Institutes of Health (NIH), Ministry of Health Malaysia, Shah Alam, 40170 Malaysia; 2 Flow Cytometry Laboratory, Biomedical Research Core Facility (BioRCF), Institute for Medical Research (IMR), National Institutes of Health (NIH), Ministry of Health Malaysia, Shah Alam, 40170 Malaysia

**Keywords:** Curcumin, Sensitisation, TRAIL, Apoptosis, TRAIL-resistant, Lung cancer

## Abstract

**Background:**

TRAIL acts as a selective anticancer agent in non-small cell lung cancer (NSCLC). However, some NSCLC are resistance to TRAIL due to the presence of several anti-apoptotic mechanisms. With the help of natural compound such as curcumin, TRAIL-resistance can be overcome and the effect of TRAIL-induce apoptosis in NSCLC can be improved. In this study, we investigated the effect of curcumin in NSCLC as sensitiser to TRAIL as well as their molecular underlying mechanism.

**Method:**

Proliferation assay was conducted to determine whether curcumin could enhance the cytotoxic effect of TRAIL in NSCLC cell lines. Flow cytometry was performed for the assessment of TRAIL receptors expression (DR4 and DR5) and apoptosis assay (Annexin-V / PI staining). Gene expression analysis using RT^2^ profiler PCR array and KEGG tools were performed to analyse the transcriptional levels of 84 significant apoptotic genes in NSCLC.

**Results:**

Treatment of NSCLC cell lines with TRAIL revealed that NSCLC cell lines can be classified as TRAIL-sensitive (H2170 and H1437) and TRAIL-resistant (A549 and H1299) based on their IC_50_ values. H1299 has slightly lower expression of DR5, as compared to the other NSCLC cell lines which have more than 90% expression of DR4 and DR5. The combined treatment of curcumin and TRAIL were able to reduce cell viability and enhanced apoptosis in both TRAIL resistant and sensitive NSCLC cells than TRAIL alone. In addition, the combined treatment seems to induce the intrinsic apoptotic pathway in P53-deficient H1299, by activating the pro-apoptotic genes which are independent of P53.

**Conclusion:**

Hence, this study provides evidence on the potential of curcumin as a sensitiser to enhance the efficacy of TRAIL in NSCLC and in TRAIL-resistance NSCLC.

**Supplementary Information:**

The online version contains supplementary material available at https://doi.org/10.1186/s12906-026-05398-z.

## Introduction

Lung cancer is the most common type of cancer in men and third in women after breast and colorectal cancer [1]. Lung cancer is also the leading cause of cancer related morbidity and mortality in men, whereas, second in women after breast cancer [2]. Non-small cell lung cancer (NSCLC) accounted for approximately 85% of all lung cancer cases. Curative surgery in NSCLC is only limited to stage 1 and 2 when the tumour is localised and has not spread to lymph nodes and other parts of the body [3]. NSCLC is also characterised by the excessive cellular proliferation and resistance to apoptosis [4]. The prognosis of NSCLC patients are poor as most conventional cytotoxic drugs and radiotherapy are unable to completely eradicate the tumour [5]. Despite several treatment modalities currently available, the 5-year overall survival for most lung cancer patient remain unchanged at about 20% and has not improved for the past decades [6].

The tumour necrosis factor (TNF) - related apoptosis inducing ligand, also known as TRAIL is a member of the TNF superfamily that induces apoptosis [7]. TRAIL binds to both death receptor 4 (DR4) and DR5 resulting in the formation of death inducing complex (DISC) which subsequently activates both caspase-8 and 3 leading to apoptosis and cell death [7]. TRAIL emerged as a promising and selective anti-cancer agent due to its specificity to target cancer cells and not normal cells [8]. Although, TRAIL has been shown effective either alone or in combination with several chemotherapeutic drugs [9, 10], many tumours including some NSCLC subtypes are resistance to TRAIL [11–13]. This is due to several factors including high expression of TRAIL-decoy receptors such as DcR1 (decoy receptor 1) or DcR2 (decoy receptor 2) which reduces the binding of TRAIL to its receptor. Moreover, the high expression of anti-apoptotic proteins such as XIAP (inhibitors of apoptosis), BCL-2 (B-cell lymphoma 2) and cFLIP (cellular FLICE-like inhibitory protein) could also interfere the process of TRAIL-induced apoptosis [14–17].

Curcumin is a natural bioactive compound of turmeric (Curcuma longa) and has been used for centuries as food and medicine. Curcumin possess several medicinal benefits including anti-oxidant and anti-inflammatory properties that can aids in the management of arthritis and hyperlipidaemia [18]. Curcumin has also been well reported for its anti-tumour properties in several pre-clinical models including breast cancer, brain tumour and lung cancer [19–21]. Furthermore, several studies have described the ability of curcumin to act as a sensitiser to cancer cells by enhancing the cytotoxicity of several chemotherapies including cisplatin [22], vinorelbine [23] and 5-fluorouracil [24]. The combination of TRAIL and curcumin have also been shown to enhance cytotoxic effect particularly in pre-clinical model of renal cell carcinoma [25], colon cancer [26] and brain tumour [27]. However, study looking into the sensitising effect of curcumin towards TRAIL-induced apoptosis in NSCLC have not been well documented. Furthermore, whether curcumin can enhance the effect of TRAIL-induced apoptosis and cellular inhibition in TRAIL-resistant NSCLC is still unknown. Therefore, the present study aims to explore the ability of curcumin to enhance the effect of TRAIL-induced apoptosis and cellular inhibition in several NSCLC cells lines including TRAIL-resistant NSCLC.

## Methodologies

### Cell culture

Four types of NSCLC cell lines (A549; adenocarcinoma, H2170; squamous cell carcinoma, H1437; adenocarcinoma and H1299; carcinoma) were used in this study. Two of these cell lines, A549 (cat no: CCL-185) and H1299 (cat no: CRL-5803) were TRAIL-resistant and the other two cell lines, H2170 (cat no: CRL-5928) and H1437 (cat no: CRL-5872) were TRAIL-sensitive. All these NSCLC cell lines were purchased from American Type Culture Collection (ATCC, Manassas, VA, USA). These cells were grown in RPMI-1640 medium containing 10% fetal bovine serum (FBS), 1% penicillin/streptomycin (all purchased from Gibco, Thermo Fisher Scientific, Inc., Waltham, MA, USA). Human adipose derived mesenchymal stem cells/MSCs (cat no: ATCC^®^ PCS-500-011) were purchased from American Type Culture Collection (ATCC, Manassas, VA, USA). The MSCs were cultured in specific growth medium containing knockout DMEM, 1% penicillin/streptomycin, 2 mM L-glutamine (200 mM stock), 10% fetal bovine serum (FBS), 5 ng/mL fibroblast growth factor (FGF) basic and 5 ng/mL recombinant epidermal growth factor (rhEGF). Cells were maintained in T75 tissue culture flasks at 37 °C in a humidified atmosphere of 5% CO_2_. Confluent cells were harvested by washing in phosphate-buffered saline (PBS) and followed by trypsinization (0.25% in EDTA) for sub-culturing.

### Inhibitory concentration (IC_50_) of curcumin or TRAIL in NSCLC cell lines

Curcumin (Sigma-Aldrich, St. Louis, MO, USA) was dissolved in a 1mL DMSO to make a stock solution of 1 M. Curcumin stock was then diluted in complete RPMI-1640 medium to provide sub-stock and final working concentrations of 4 ng/mL, 8 ng/mL, 12 ng/mL, 15 ng/mL, 20 ng/mL, 22 ng/mL and 26 ng/mL. Recombinant human TRAIL (rhTRAIL; cat no: PHC 1634) (Thermo Fisher Scientific, Inc., Waltham, MA, USA) was purchased as 50 µg stock and diluted in DPBS for a working concentration of 500 ng/mL, 250 ng/mL, 125 ng/mL, 60 ng/mL, 30 ng/mL, 15 ng/mL and 7.5 ng/mL. The IC_50_ of NSCLC cell lines treated with either curcumin or recombinant human TRAIL (rhTRAIL) was assessed by the MTS assay [3-(4,5-dimethylthiazol-2-yl)-2 H-tetrazolium, inner salt] purchased from Promega (CellTiter 96^®^ AQueous One Solution Cell Proliferation Assay, Madison, WI, USA). NSCLC cells and MSCs were plated at a concentration of 5.0 × 10^3^ cells/well in 96 wells plate and incubated overnight in humidified air with 5% CO_2_ at 37 °C. Cells were then treated with 100 µL working concentration of curcumin (4 ng/mL, 8 ng/mL, 12 ng/mL, 15 ng/mL, 20 ng/mL, 22 ng/mL and 26 ng/mL.) or rhTRAIL (of 500 ng/mL, 250 ng/mL, 125 ng/mL, 60 ng/mL, 30 ng/mL, 15 ng/mL and 7.5 ng/mL) for 48 h. After 48 h incubation, 10 µl of MTS solution was added to each well and incubated for another 4 h. Finally, absorbance was measured using Envision 2103-0020 plate reader at 490 nm (Perkin Elmer, Waltham, MA, USA), using wells without cells as blank. Cells viability was calculated according to the following formula: $\text{Cell viability}\left(\%\right)=\mathrm{Cells}\left (\mathrm{sample}\right)/\mathrm{Cells} \left(\mathrm{control}\right)\times100$ and IC_50_ was calculated using log formula.  

### Proliferation assay (Combined effect of Curcumin and TRAIL)

The proliferation assay was used to evaluate whether curcumin enhances the cytotoxic effect of TRAIL or acts as a sensitiser. Cells were seeded at 5.0 × 10³ cells/well in a 96-well plate and incubated overnight in humidified air with 5% CO₂ at 37 °C. The next day, medium was discarded and replaced with either single treatment of curcumin or TRAIL or the combination of both for 48 h. The concentration of treatment was based on the IC_50_ values of both curcumin and TRAIL. Following incubation, 20 µL of MTS solution (CellTiter 96^®^ AQueous One Solution Cell Proliferation Assay, Promega, Madison, WI, USA) was added into the wells and incubated for 4 h. After the incubation period, absorbance was assessed using an Envision 2103-0020 plate reader at 490 nm (Perkin Elmer, Waltham, MA, USA) and cell viability was calculated using the formula mentioned earlier.

### Proliferation assay (Sensitising Effect of Curcumin to TRAIL)

Analysis of sensitising effect of curcumin was performed by initially seeded 1.0 × 10^6^ cells in 5 mL of curcumin (IC_50_ values according to cell lines) in T25 flask for 24 h in humidified CO_2_ incubator with 5% CO_2_ at 37 °C. After 24 h of incubation, cells were harvested by trypsinisation and re-seeded at 5.0 × 10^3^ cells/well in 96-well plate for another 24 h with different concentrations of TRAIL (500 ng/mL, 250 ng/mL, 125 ng/mL, 60 ng/mL, 30 ng/mL, 15 ng/mL, 7.5 ng/mL 4ng/mL and 2 ng/ml) and incubated for another 24 h. Following incubation, 20 µL of MTS (CellTiter 96^®^ AQueous One Solution Cell Proliferation Assay, Promega, Madison, WI, USA) was added into the wells and incubated for 4 h. Absorbance was assessed using an Envision 2103-0020 plate reader at 490 nm (Perkin Elmer, Waltham, MA, USA) and cell viability was calculated using the formula mentioned earlier.

### Analysis of DR4 and DR5 in NSCLC cell lines

The NSCLC cell lines were analyzed for TRAIL receptors expression (DR4 and DR5) with Phycoerythrin (PE)-conjugated TRAIL-R1 (DR4) (cat no: 12-9927-42) or PE-conjugated TRAIL-R2 (DR5) (cat no: 12-9908-42) (Thermo Fisher Scientific, Inc., Waltham, MA, USA) using flow cytometry (Becton Dickinson BD). Briefly, NSCLC cells (1.0 × 10^6^ cells in 200 µL of DPBS) were stained with the antibodies (5 µL of DR4 or DR5-PE) for 30 min on ice in the dark. After the washing steps, stained cells were subjected to flow cytometric analysis using the FACS Calibur instrument (Becton Dickinson BD), and a total of 10,000 events were acquired and analyzed using Cell Quest software (Becton Dickinson BD).

### Annexin-V and propidium iodide (PI) assay

Apoptosis was evaluated using FITC-conjugated annexin V antibody from BD Pharmingen (Becton Dickinson Biosciences, San Jose, CA, USA). In brief, NSCLC cell lines (A549, H2170, H1437 and H1299) were initially seeded (5.0 × 10^5^cells in 1.0 mL of medium) in a 6-well tissue culture plate. Next, treatments of either curcumin (IC_50_ values according to cell lines), TRAIL (IC_50_ values according to cell lines) or the combination of both were added to the wells. After 24 h, cells were harvested by trypsinization and collected by centrifugation. Annexin V binding buffer (100 µL) was added with 1 µL of annexin V–FITC antibody and incubated at 4 °C in the dark. For dead cell analysis, 1 µL of propidium iodide (PI) (Invitrogen, Thermo Fisher Scientific, Inc., Waltham, MA, USA) was added to 500 µL of cell suspension and directly used for analysis. Stained cells were subjected to flow cytometric analysis, and 10,000 events were collected using the FACS Calibur instrument (Becton Dickinson BD) and analyzed using Cell Quest Pro software gated on FL-1 (Becton Dickinson BD).

### RT^2^ profiler PCR arrays

The 84 key genes and apoptotic pathways were analysed in TRAIL-resistance H1299 using the Apoptosis RT^2^ Profiler PCR Array (PAHS-012Z; Qiagen, MD, USA). The human RT² profiler PCR array (apoptosis) profiles the transcriptional level using quantitative RT-PCR of 84 critical genes categorically divided into anti-apoptosis, regulators of apoptosis, death domain receptors, and caspases. TRAIL-resistance H1299 was treated with either curcumin (15ng/mL), TRAIL (100ng) or the combination of both for 48 h. Total RNA was extracted from samples and on-column DNA digestion was performed during the extraction process. RNA (0.5 µg) was added into the genomic DNA elimination mix to a total of 10 µL. The sample was incubated at 42 °C for 5 min and then on ice for 1 min. Reverse transcription mix (10 µL) was then added, and the sample was incubated at 42 °C for 15 min and 95 °C for 5 min. After the reaction time, 91 µL of RNAse-free water was added to make a total of 111 µL of cDNA synthesizing reaction. The PCR component mix was prepared by adding 102 µL of cDNA synthesizing reaction to 2× RT^2^ SYBR Green Mastermix and RNase-free water to a total volume of 2700 µL. Subsequently, 25 µL of the reaction was pipetted into each well of the 96-well PCR array plate and centrifuged at room temperature for 1 min at 1000× g (all reagents were purchased from Qiagen, MD, USA). The PCR cycling process (one cycle at 95 °C for 10 min, followed by 40 cycles of 95 °C for 15 s and 60 °C for 60 s) was performed using Applied Biosystems™ (ABI) StepOne™ Real-Time PCR System (Fisher Scientific, Hampton, New Hampshire, United States). Analyses of data were performed using Qiagen web-based analysis tools (https://geneglobe.qiagen.com/my). Genes that were up-regulated or down - regulated between the combined treatment versus control (untreated) analyzed using the R studio (Northern Ave, Boston, MA, USA) and its built-in Kyoto Encyclopedia of Genes and Genomes (KEGG) pathway analysis.

### Statistical analysis

Data are presented as mean ± standard deviation (SD) of three independent experiments. Statistical analyses were performed using either two-tailed t-test (for comparison between two groups) or one-way analysis of variance (ANOVA) for comparison between groups followed by Dunnett’s post-hoc test. Analyses were conducted using Microsoft Excel 2010 (version 14.0; Microsoft Corporation, Redmond, WA, USA) and GraphPad Prism (version 10.6.0) with p-value of < 0.05 considered as statistically significant.

## Results

### TRAIL-sensitive and TRAIL-resistant NSCLC cell lines

**Fig. 1:**
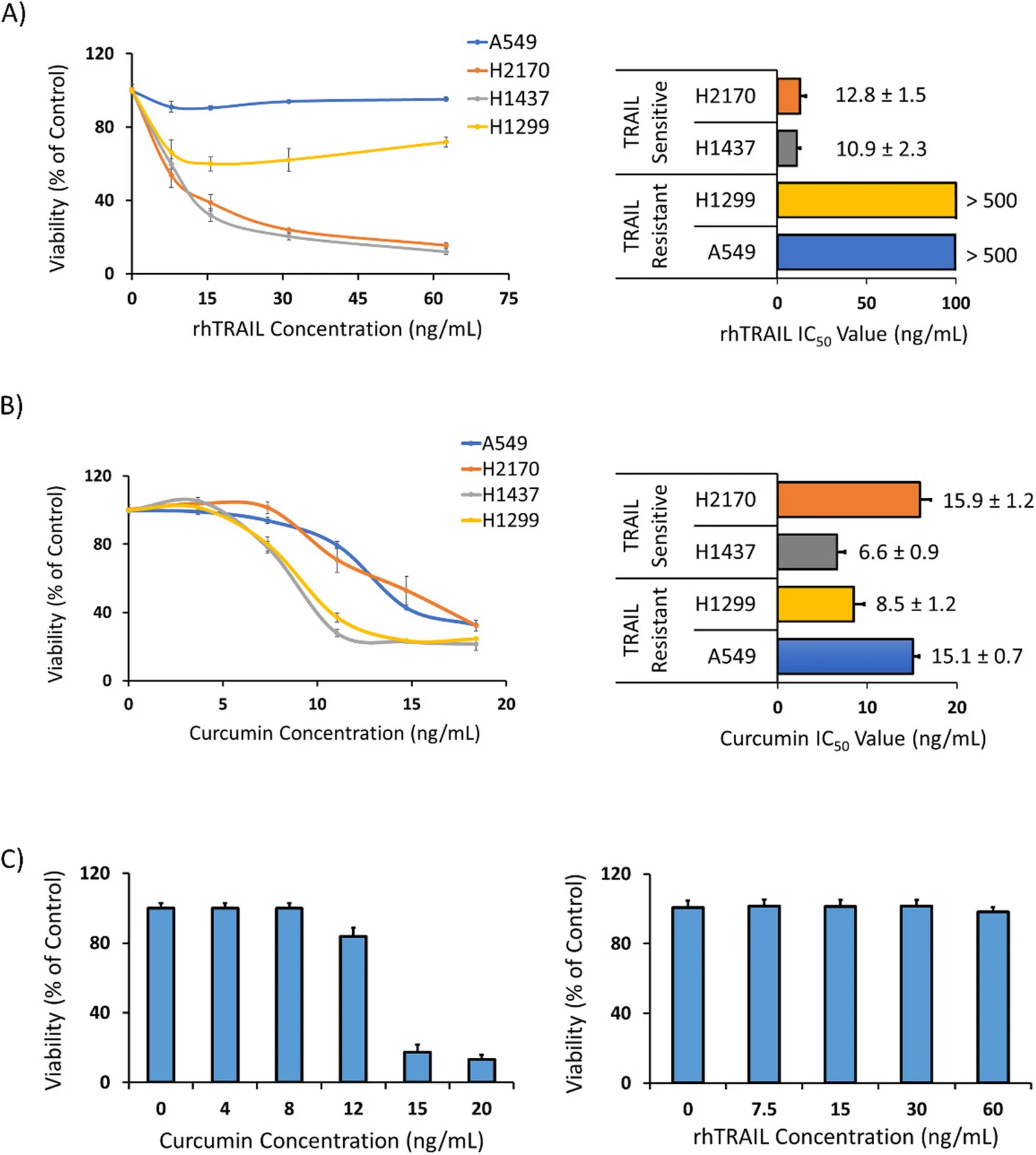
Effect of Curcumin and rhTRAIL on the proliferation of NSCLC cell lines and MSCs. **A** Treatment of NSCLC cell lines using rhTRAIL shows that the NSCLC cell lines can be categorised as TRAIL-sensitive or TRAIL-resistant according to its IC_50_ values. **B** TRAIL-sensitive and -resistant NSCLC cell lines were treated with different concentration of curcumin and the IC_50_ values were calculated for each cell lines. **C** The effect of curcumin or TRAIL in MSCs was analysed as a model for toxicity

To characterise the sensitivity of NSCLC cell lines to TRAIL, cells were treated with increased concentration of recombinant TRAIL (rhTRAIL) for 48 h and subjected to proliferation assay. Analysis using viability assay indicated that each type of NSCLC cell lines exhibits different sensitivity to the rhTRAIL (Fig. 1A, left panel). Based on the calculated IC_50_ values, H2170 and H1437 were highly sensitive to rhTRAIL and categorised as TRAIL-sensitive, while H1299 and A549 were highly resistance to the effect of TRAIL and categorised as TRAIL-resistant cell lines (Fig. 1A, right panel).

### Curcumin inhibit growth of NSCLC cell lines

Curcumin has drawn particular interest in cancer research due to its anti-cancer properties and chemo-sensitising effect. However, the exact mechanism on how curcumin can increase the sensitivity of tumour cells to chemotherapies is not yet fully understood. To evaluate the combination effect of curcumin and TRAIL in NSCLC, the IC_50_ values of curcumin was initially evaluated in both TRAIL-sensitive and TRAIL-resistant NSCLC cell lines using proliferation assay. All the NSCLC cell lines were sensitive to curcumin; however, the degree of sensitivity varies between the NSCLC cell lines (Fig. 1B, left panel). Two of the NSCLC cell lines, H1437 and H1299 were more sensitive to curcumin compared to A549 and H2170. The IC_50_ values of curcumin were also calculated for each NSCLC cell lines. (Fig. 1B, right panel).

### Cytotoxicity of curcumin and TRAIL in normal cells

Human MSCs was used to analyse the effect of TRAIL and curcumin in normal cells. High concentration of curcumin between 15 and 20 ng/mL inhibited the proliferation of MSCs while concentration of curcumin lower than 12 ng/mL did not have any effect to inhibit the cells (Fig. 1C, left panel). MSCs were noticed resistance to the effect of TRAIL-induced inhibition as cells were not inhibited when cultured with the rhTRAIL with any of the given concentrations (Fig. 1C, right panel).

### TRAIL-receptors expression (DR4 and DR5) in NSCLC

**Fig. 2:**
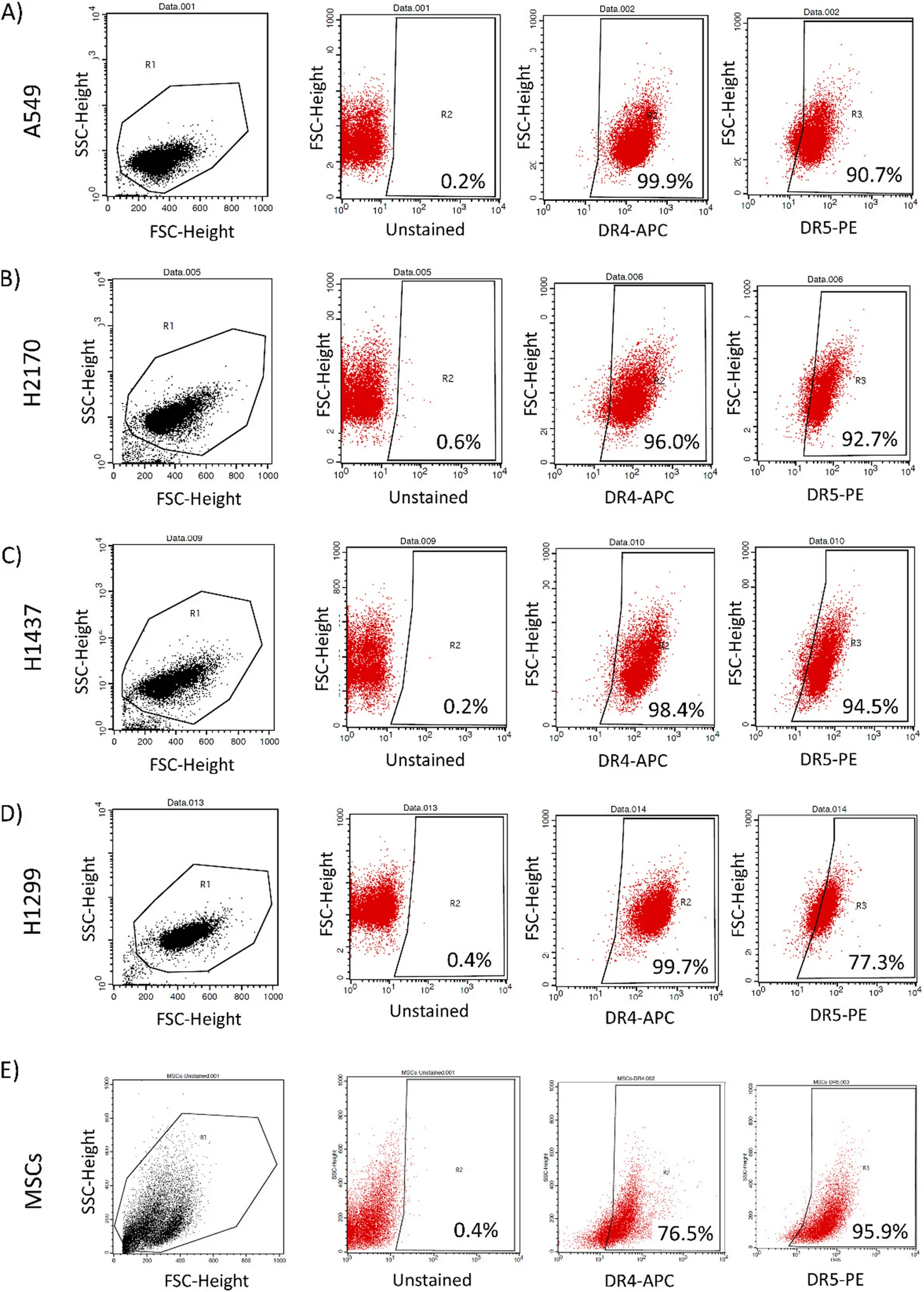
Expression of DR4 and DR5 in NSCLC cell. **A-E** Representative dot plot showing the expression of DR4 and DR5 in NSCLC cell lines and MSCs. Forward (FSC) and side scatter (SSC) gating were used to remove dead cells and isotype control was used to gate out unstained cells. Apart from a slightly low expression of DR5 noticed in H1299, all of the other NSCLC cell have elevated level of DR4 and DR5 with more than 90% expression

The expression of TRAIL-receptors (DR4 and DR5) could influence the sensitivity of NSCLC cell to TRAIL-induced inhibition as studies suggested. In this analysis, the expressions of TRAIL-receptors were analysed using PE-conjugated antibodies against DR4 and DR5. Flow cytometry analyses revealed that all four NSCLC cell lines expressed more than 95% of DR4 receptor (Fig. 2). However, a slightly lower DR5 expression (77.0 ± 0.5%) was noticed for H1299 cells as compared the other three NSCLC cell lines, A549, H2170 and H1437 that have more than 90% expression (Fig. 2).

### Combined effect of TRAIL and curcumin on TRAIL-resistant NSCLC

**Fig. 3:**
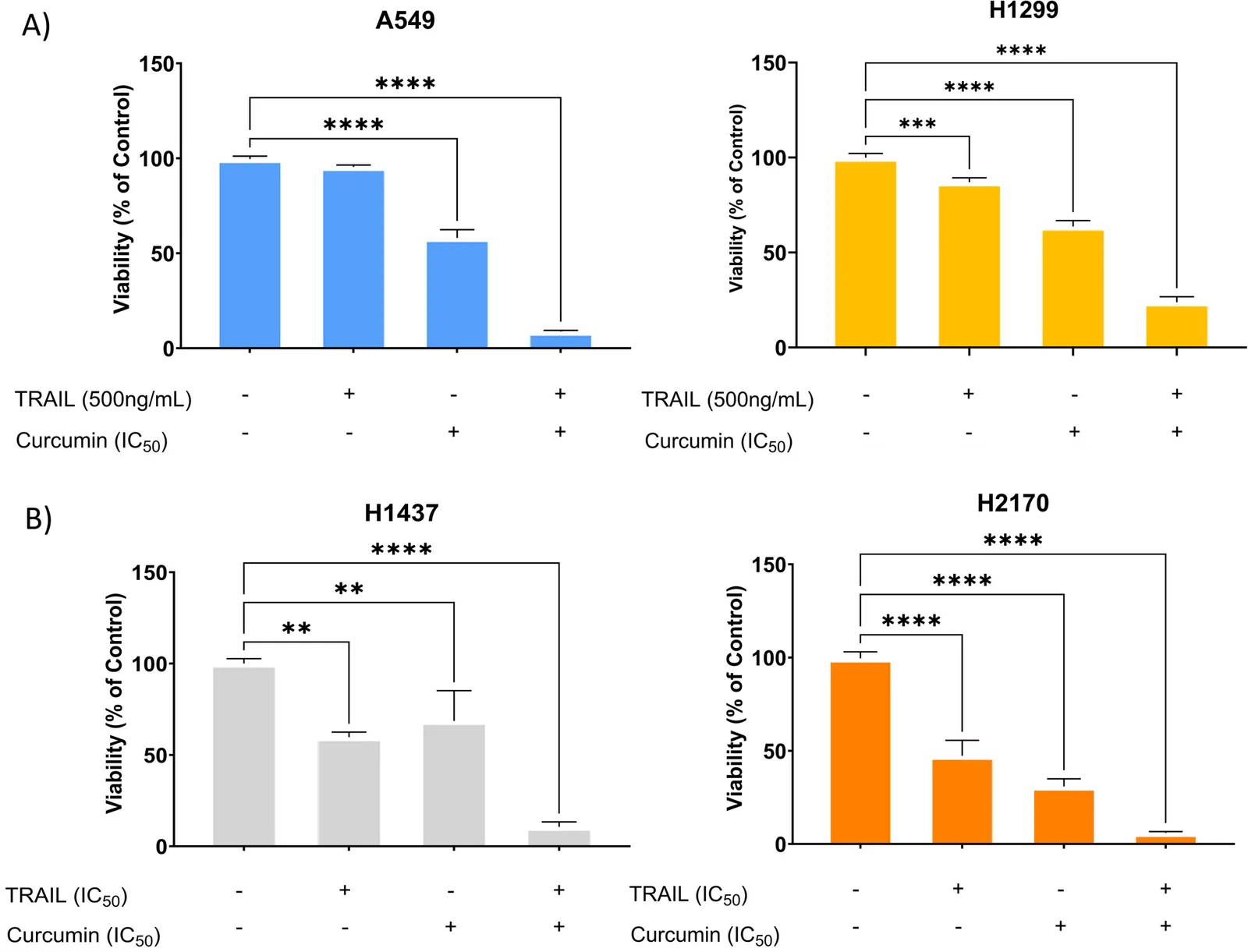
The effect of TRAIL, Curcumin or Combination of Both on TRAIL-Resistant (A549 and H1299) and TRAIL-Sensitive (H1437 and H2170) NSCLC. **A** TRAIL-resistant NSCLC cells were treated with the highest concentration of TRAIL (500ng/mL), curcumin (IC_50_) or the combination of both for 48 h. When compared to either TRAIL or curcumin alone, the combined treatment noticeably able to reduce the percentage of viability in both A549 and H1299 cells. **B** TRAIL-sensitive NSCLC were treated with the IC_50_ values of either TRAIL, curcumin, or the combination of both for 48 h. Significant inhibition of cell viability can be noticed for the combined treatment in both H1437 and H2170 as compared to TRAIL or curcumin alone (one-way ANOVA followed by Dunnett’s post hoc test; **p < 0.001)

The combined effect of TRAIL and curcumin to inhibit cell viability of TRAIL-resistant NSCLC were evaluated by culturing these cell lines with rhTRAIL, curcumin or the combination of both for 48 h and subjected to proliferation assay (Fig. 3). Treatment of using the highest concentration of TRAIL (500ng/ml) in both A549 and H1299 did not inhibit the proliferation of these two cell lines indicating that both A549 and H1299 were TRAIL-resistant (Fig. 3A). Furthermore, treatment of using curcumin alone was only able to inhibit 50% of cell viability in both A549 and H1299 (Fig. 3A). However, when both TRAIL and curcumin were combined, a significant inhibition of cell viabilities in both A549 (8.76 ± 1.6%) and H1299 (23.8 ± 2.9%) were noticed compared to TRAIL (A549; 94.8 ± 1.8% and H1299; 87.0 ± 2.4%) or curcumin (A549; 58.1 ± 4.4 and H1299; 63.7 ± 3.1%) alone respectively (Fig. 3A). Treatment of using rhTRAIL or curcumin resulted in 50% inhibition in H1437 and H2170 cell viabilities as compared to without treatment as shown in Fig. 3B. Moreover, the combination of both TRAIL and curcumin significantly inhibited the proliferation of both H1437 and H2170 with only 13.2 ± 10.7% and 5.9 ± 0.7% cell viability detected for both cells respectively (Fig. 3B).

### Curcumin sensitised TRAIL-resistant NSCLC to TRAIL-induced inhibition

**Fig. 4:**
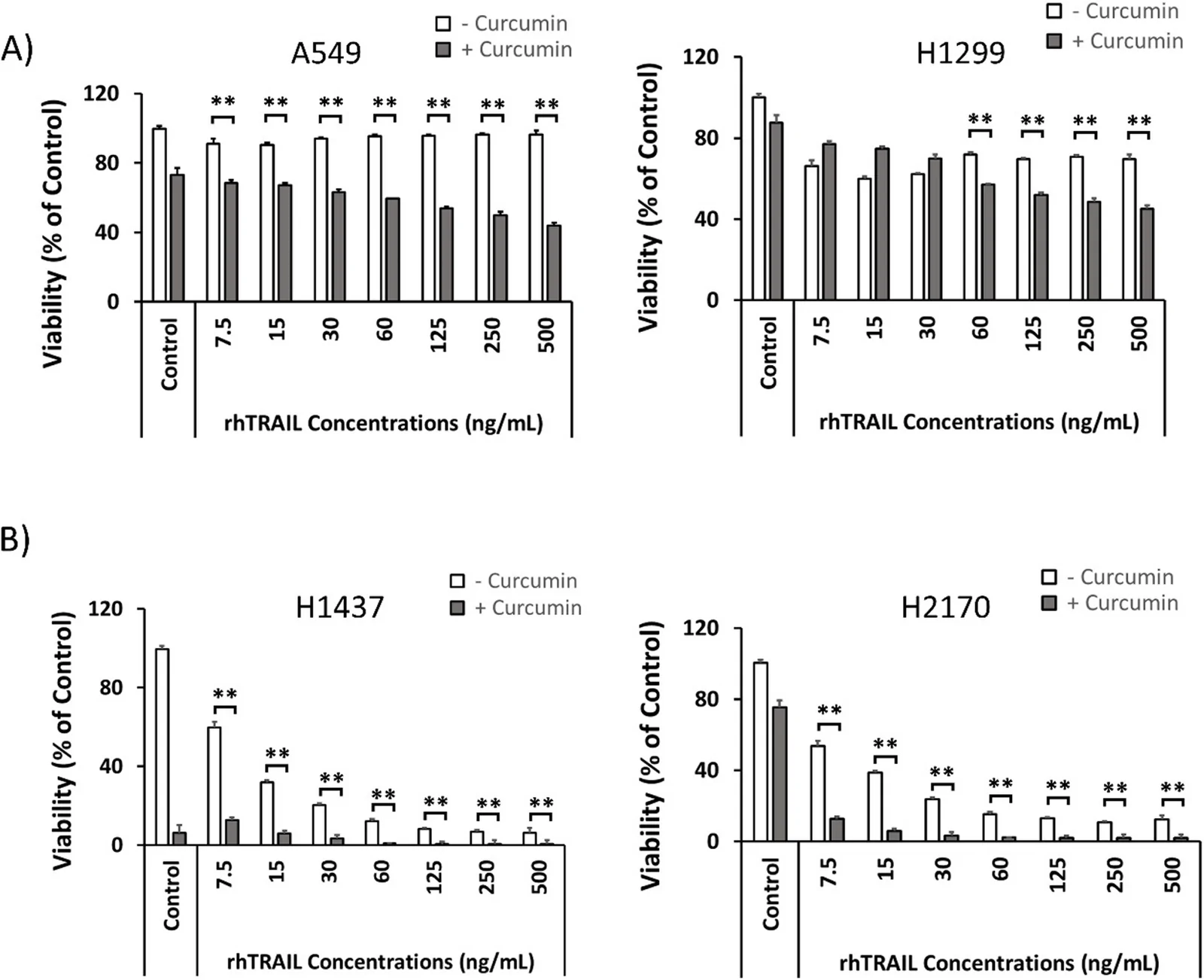
The Effect of Curcumin Sensitisation on the Viability of TRAIL-resistant NSCLC Cells. **A** TRAIL-resistant NSCLC cells were initially treated with curcumin for 24 h, followed by rhTRAIL for another 24 h and subjected to proliferation assay. Curcumin treatment prior to rhTRAIL markedly reduce the percentage of cell viability in A549 cell for all the rhTRAIL concentrations; however, the sensitising effect of curcumin in H1299 was prominent only when 60ng/ml or higher concentration of rhTRAIL was used. **B** Analysis of the effect of curcumin prior to rhTRAIL in TRAIL-sensitive cells. Inhibition of cell viability can be noticed in all the rhTRAIL concentrations and the inhibition was significantly greater when curcumin was introduced prior to rhTRAIL (t-test; **p < 0.001)

The sensitising effect of curcumin on TRAIL-resistant NSCLC was evaluated in this study. The NSCLC cell lines were pre-treated with curcumin using the IC_50_ values for 24 h. Next, the cells were harvested and re-seeded with different concentrations of rhTRAIL for another 24 h and subsequently analysed for its viability. Treatment up to 500ng/mL of rhTRAIL has no significant effect to inhibit the viability of both A549 and H2199 cells indicating that these cells were TRAIL-resistant (Fig. 4A). Sensitisation using curcumin prior to rhTRAIL was able to significantly reduced cell viability in the A549 cells compared to rhTRAIL alone (Fig. 4A; left panel). Sensitisation of using curcumin seems to only works when in combination with rhTRAIL at a concentration of 60ng/mL or higher as noticed for the H1299 cell (Fig. 4A; right panel). Treatment of using rhTRAIL from the lowest concentration of 7.5ng/mL to the highest concentration of 500ng/mL was effective to inhibit the viability of both H1437 and H2170 signifying that these two cells were TRAIL-sensitive (Fig. 4B). Furthermore, significant inhibition of cell viability was detected in both H1437 and H2170 when curcumin was used prior to rhTRAIL in all the concentrations (Fig. 4B).

### Combined treatment enhanced TRAIL-induced apoptosis in NSCLC

**Fig. 5:**
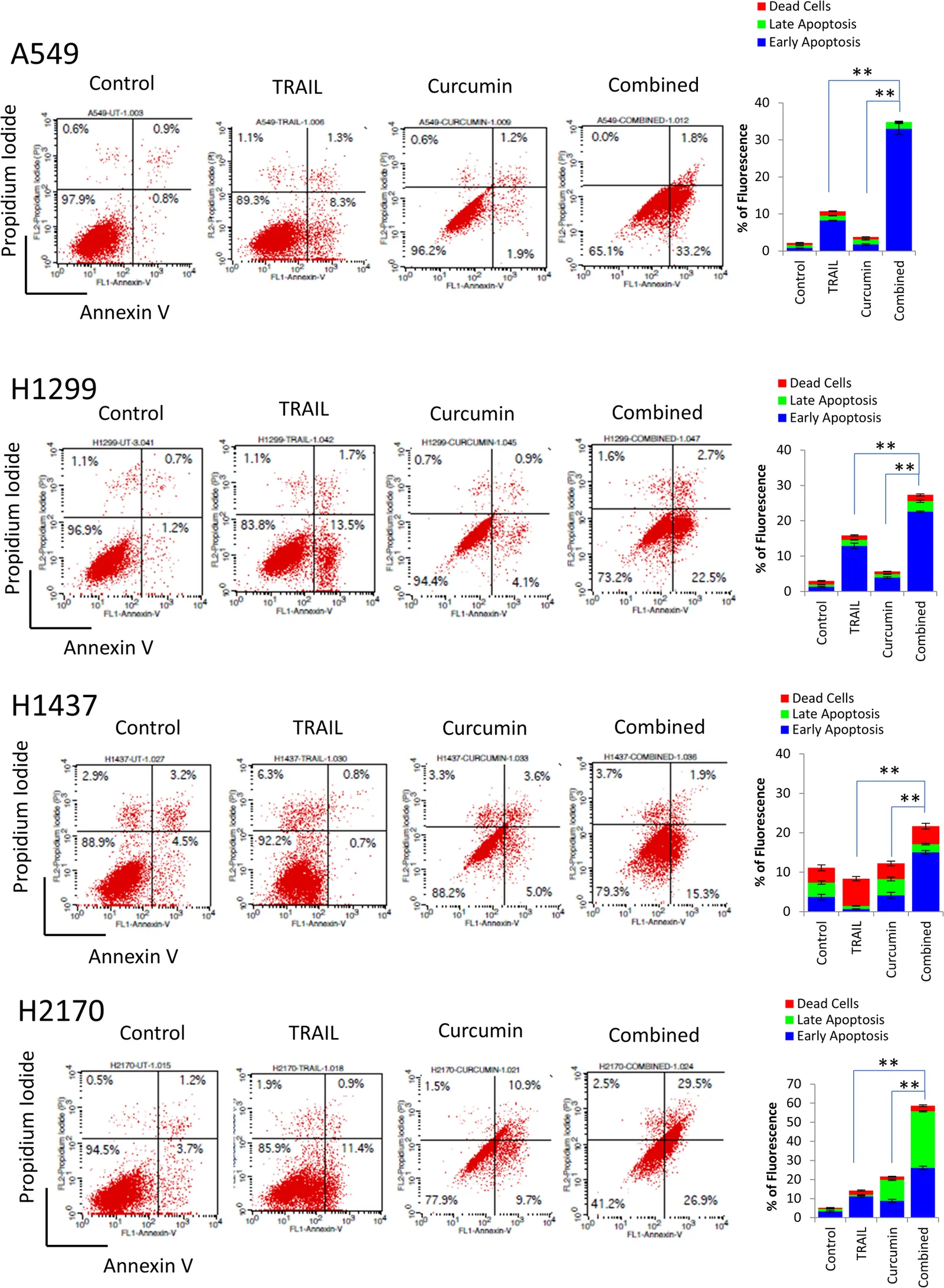
Analysis of Apoptosis in NSCLC cells. NSCLC cells were initially treated with curcumin for 24 h followed by rhTRAIL for another 24 h and subsequently stained with Annexin V and PI and were subjected to flowcytometry analysis. Bottom right quadrant: positive for Annexin V (early apoptotic cells); top right quadrant: positive for both PI and Annexin V (late apoptotic/necrotic secondary necrosis); top left quadrant: positive for PI (dead cells); bottom left quadrant: negative for both Annexin V and PI (viable cells). Percentage of cell populations (early apoptotic, late apoptotic, and dead cells) were also analyzed in triplicates and depicted as cumulative bar charts to show the total cell death induction (t-test; **p < 0.001).

Flow cytometry was conducted using Annexin V (AV) and PI double staining to determine the degree of apoptosis using either single treatment of rhTRAIL or curcumin or the combination of both in NSCLC cells (Fig. 5). The results showed that the basal expression of AV (control) was low for all the untreated NSCLC cell indicated that many cells were alive in the untreated control (A549; 0.8 ± 1.5%, H1299; 1.3 ± 1.8%, H1437; 3.7 ± 1.7%, H2170; 3.3 ± 1.4%). Findings indicated that following 24 h exposure to rhTRAIL, there was a slight increase in the percentage of early apoptosis in the NSCLC cell with 8.2 ± 0.5% detected in A549, 12.9 ± 0.8% in H1299, 0.7 ± 0.1% in H1437 and 11.2 ± 0.3% in H2170. There was also a small increase of early apoptosis detected in the NSCLC cell lines after treatment of using curcumin (A549; 1.73 ± 0.2%, H1299; 4.0 ± 0.2%, H1437; 4.1 ± 0.8%, H2170; 8.8 ± 0.8%; respectively). Interestingly, the combined treatment of curcumin and TRAIL significantly increased the percentage of early apoptosis in the TRAIL-resistant NSCLC cell as compared to rhTRAIL alone with 32.9 ± 1.5% and 22.6 ± 0.5% of Annexin V-positive detected in in A549 and H1299 respectively. Similarly, a higher percentage or apoptosis was also detected in TRAIL- sensitive NSCLC as compare to TRAIL alone with 15.0 ± 0.5% and 26.1 ± 0.9% of Annexin V-positive detected in H1437 and H2170 correspondingly.

### Analysis of apoptotic genes in H1299 though combined treatment

**Fig. 6:**
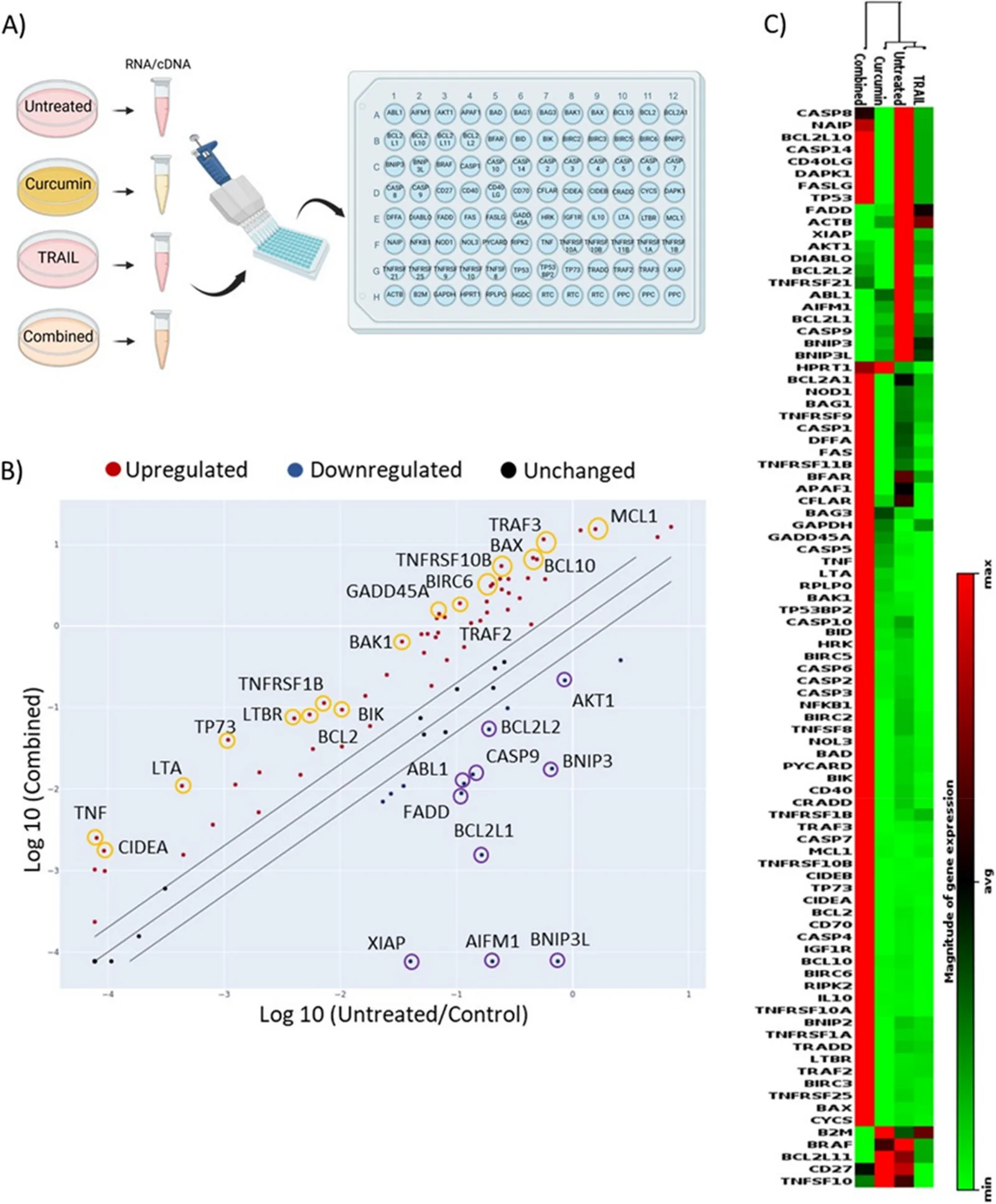
Analysis of apoptotic genes in TRAIL-resistance H1299 regulated by the combined treatment of curcumin and TRAIL. **A** Schematic diagram of each sample, including cDNAs and primers of each individual gene pre-casted inside a 96-well plate. **B** Regulated genes (upregulated or downregulated) in TRAIL-resistance H1299 treated with the combined treatment of TRAIL and curcumin. **C** Hierarchical cluster of TRAIL-resistance H1299 treated with curcumin, TRAIL, or the combination of both and untreated as control

Pathway-specific gene expression analysis was performed using RT^2^ profiler PCR arrays (Qiagen, Hilden, Germany) to identify apoptotic genes that are uniquely regulated in TRAIL-resistant H1299 cell line upon treatments. H1299 was subjected to TRAIL, curcumin, or the combination of both for 48 h, followed by RNA extraction and cDNA synthesis. Synthesized cDNA from each sample was pipetted into a pre-casted 96-well plate containing primers specific for each individual apoptotic gene. Subsequently, quantitative PCR was performed using Applied Biosystems™ StepOne™ Real-Time PCR System (Fisher Scientific, New Hampshire, United States). The Ct values were uploaded into the Gene Globe Data Analysis Centre (Qiagen) and later analysed using the Qiagen web-based tools (https://geneglobe.qiagen.com/my/analyze) (Fig. 6A). Scatter plotanalysis showed that the combined treatment of using curcumin and TRAIL, increased the expression of several apoptotic genes (highlighted orange), and reduce the expression of some other genes (highlighted purple) in TRAIL-resistant H1299 as compared to untreated (control) (Fig. 6B). From all the 84 genes analysed in the combined treatment, no less than 17 genes including *MCL1*,* BCL10*,* TRAF3*,* BAX*,* TNFRSF10B*,* BIRC6*,* GADD45A*,* TRAF2*,* BAK1*,* TNFRSF1B*,* BIK*,* BCL2*,* LTBR*,* TP73*,* LTA*,* CIDEA* and *TNF* were extremely upregulated, and 10 genes were highly downregulated including *ABL1*,* FADD*,* XIAP*,* AIFM1*,* BNIP3L*,* BCL2L1*,* BNIP3*,* CASP9*,* BCL2L2* and *AKT1* (Fig. 6B). In the resulting cluster gram, the upregulated genes appear in red, the downregulated in green while the non-affected appear in black. Samples from untreated (control), curcumin or TRAIL tend to cluster together, while the combine treatment (curcumin and TRAIL) shown to have a slightly different profile (Fig. 6C).

### KEGG pathway analysis of apoptosis

**Fig. 7:**
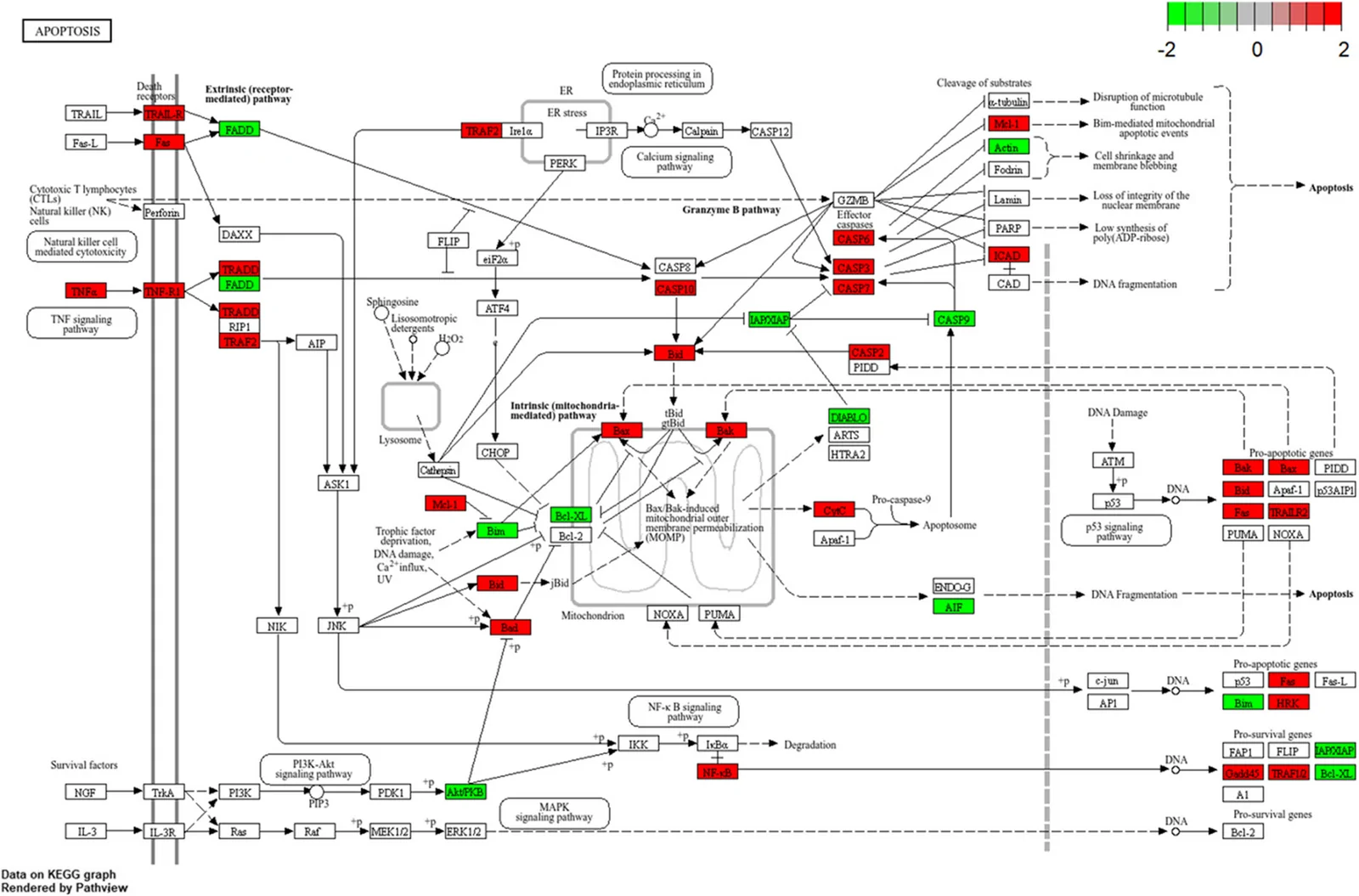
Kyoto Encyclopaedia of Genes and Genomes (KEGG) analysis of apoptosis pathway in H1299 treated with the combination of curcumin and TRAIL. Genes that are shown in the red boxes are up-regulated and those in green boxes are down-regulated in H1299. Note that those presented within white boxes are unchanged apoptotic genes

To understand further the functional relationship between these apoptotic genes, we conducted KEGG pathway analysis using the R studio (Fig. 7). The KEGG pathways analysis showed that the altered genes were part of the core network of genes that drives apoptosis. The genes upregulated in H1299 upon treatment with curcumin and TRAIL included mostly the pro-apoptotic genes corresponding mainly to the intrinsic (mitochondria-mediated) and extrinsic apoptotic pathway (Fig. 7).

## Discussion

Several studies have shown that sensitisation using first line chemotherapies as pre-treatment were able to enhance the effect of TRAIL-induced apoptosis in lung cancer cells, including several TRAIL-resistant cell lines [28–30]. However, as most chemotherapies are generally expensive, we seek to find an alternative to chemotherapies using natural compound such as curcumin. Although there are several options of chemotherapies available, highly effective, and inexpensive chemotherapy approaches for treating NSCLC are still needed. Therefore, using curcumin as an alternative to chemotherapy may provide a platform for the development of new and improved strategies for treating NSCLC. Furthermore, curcumin is considered inexpensive, readily available, and most likely to have fewer side effects as compared to conventional chemotherapies. For this report, we show for the first time that curcumin, a compound extracted from turmeric or Curcuma longa L., could be used as a potential chemo-sensitiser able to enhance the effect of TRAIL in TRAIL-resistant cell lines.

Treatment of NSCLC cell lines (H2170, H1437, H1299 and A549) using increased concentration of TRAIL (up to 100ng/mL) resulted in the separation of NSCLC cell lines into two categories of either TRAIL-sensitive or TRAIL-resistant cell lines (Fig. 1A). Recombinant TRAIL (rhTRAIL) was highly effective to inhibit TRAIL-sensitive cell lines as shown by the low IC_50_ values in both H2170 (12.8 ± 1.5 ng/mL) and H1437 (10.9 ± 2.3 ng/mL) respectively (Fig. 1A). However, treatment up to 100ng/mL of rhTRAIL was unable to inhibit the proliferation of both A549 and H1299 suggesting that these two cell lines are highly resistant to TRAIL (Fig. 1A). Although the exact mechanisms that contribute to the characteristics of TRAIL-resistance in A549 and H1299 have not been fully elucidated, studies suggested that mutation of the KRAS (Ki-ras2 Kirsten rat sarcoma viral oncogene homolog) in A549 and deficient of P53 (Tumor protein P53) in H1299 might leads to a higher cellular proliferation in these two cell lines and contributed to the characteristic of TRAIL-resistant in both A549 and H1299 [31, 32].

Curcumin was highly effective to inhibit both TRAIL-sensitive and TRAIL-resistance NSCLC cell lines with an average IC_50_ values ranging from 6.6 ± 0.9 to 15.9 ± 1.2 ng/mL (Fig. 1B). For TRAIL-resistance cell lines such as H1299 and A549, curcumin could serve as an effective adjuvant therapy as it can modulate the activity of several signalling and transcription molecules leading to a greater sensitivity of these cells to TRAIL [33]. Curcumin may also induce autophagy, or self-eating mechanism, making it an effective anti-cancer compound in NSCLC [34]. Although curcumin has been shown by several reports to be quite safe, we have noticed that at a concentration of 12 ng/mL, curcumin could inhibit MSCs proliferation suggesting that at a certain dosage (e.g. >12ng/mL), curcumin might be toxic to normal cells (Fig. 1C; left panel). Analysis on the effect of TRAIL in normal cell (MSCs) showed that treatment of up to 60 ng/mL of rhTRAIL was unable to induce any effects indicating that TRAIL is relatively safe and would not cause damages to normal cells (Fig. 1C; right panel). Therefore, further evaluation using lung-specific normal cell models may further strengthen safety assessment.

TRAIL induces apoptosis through binding to its specific death receptors, DR4 and DR5 express mostly on the surface of cancer cells [35]. Analysis on the expression of DR4 and DR5 in NSCLC indicated that the expressions of both receptors were generally high in the NSCLC cell lines. However, a slightly lower expression of DR4 in MSCs and DR5 in H1299 were noticed, suggesting that the characteristic of TRAIL-resistance as seen in both cell types might be due to the low expression of either one of these receptors (Fig. 2). While high expression of the death receptors DR4 and DR5 might suggest sensitivity to TRAIL, many cancer cells with a high level of these receptors are resistant to TRAIL even. This resistance can stem from several factors, including receptor dysfunction or defects in the FADD formation that can interfere with the TRAIL signalling activation [36, 37]. Although the expression of DR4 and DR5 were evaluated in the NSCLC cell lines, additional functional studies such as receptor blocking or silencing could further confirm their role as modulator for TRAIL-sensitivity during curcumin sensitization. In addition, curcumin-mediated sensitisation has been reported to involve reactive oxygen species (ROS) generation and endoplasmic reticulum (ER) stress, which may lead to modulation of death receptor expression. However, these mechanisms were not investigated in the present study and should be addressed for future investigation.

Although both A549 and H1299 were resistance to TRAIL, the combination of both curcumin and TRAIL have greatly improved the inhibition of TRAIL-resistant A549 and H1299 cell lines indicates an enhanced anti-tumour activity of both agents (Fig. 3A). Similar observation was noticed for the TRAIL-sensitive cell lines, whereby a significant inhibition of more than 90% cell viability were observed in both H1437 and H2170 treated with both curcumin and TRAIL indicating potential effectiveness of both agents for NSCLC treatment. This is particularly significant as TRAIL resistance in NSCLC is frequently associated with elevated levels of anti-apoptotic proteins such as BCL-2 and BAX. Curcumin may counteract this resistance by modulating the expression of these proteins [38].

Sensitization with curcumin prior to TRAIL significantly inhibited the proliferation of TRAIL-resistant NSCLC cell lines (A549 and H1299) compared to TRAIL alone. However, achieving over 50% cellular inhibition in these resistant cells required high concentration of TRAIL (Fig. 4A). This resistance is likely due to mechanisms such as defects in TRAIL signalling pathways or overexpression of anti-apoptotic proteins, which decrease the sensitivity of these cells to TRAIL-induced apoptosis. We hypothesize that by extending the sensitization period from 24 h, as performed in this assay, to 48 h could further enhance TRAIL sensitivity in these resistant cell lines [30]. Nonetheless, our findings confirm that the combined treatment of curcumin and TRAIL effectively induces apoptosis in both TRAIL-sensitive and TRAIL-resistant NSCLC cells as shown in Fig. 4A and B.

The combined treatment of curcumin and TRAIL significantly induced apoptosis in both TRAIL-sensitive and TRAIL-resistant NSCLC cell lines compared to single treatments (curcumin or TRAIL) alone (Fig. 5). While the exact mechanism by which curcumin enhances the effect of TRAIL remains unclear, several studies have suggested that curcumin may modulate the extrinsic apoptotic pathway through upregulation of TRAIL receptors and activation of caspases [30, 39]. Furthermore, Yang et al. reported that curcumin could enhance TRAIL-induced apoptosis in non-small cell lung cancer cells through inhibition of reactive oxygen species (ROS)-mediated mitochondrial pathways [40]. Similarly, Jung et al. demonstrated that curcumin sensitized human renal cancer cells to TRAIL-induced apoptosis by regulating death receptors and modulating the intrinsic apoptotic pathway through mitochondrial signaling [25, 29].

The expression of BCL-2–associated X protein or BAX was found to be low in the untreated H1299 as compared to the single treatment alone (curcumin or TRAIL) or the combination of both. Since BAX helps with the release of cytochrome c, which in turn activates caspases and subsequently apoptosis, low expression BAX in the untreated H1299 indicated that these cells are apoptosis-resistant [41, 42]. However, when H1299 cells were treated with the combination of curcumin and TRAIL, the expression of BAX was noticeably higher compared to TRAIL alone, suggesting that the combined treatment may enhance the induction of apoptosis in TRAIL-resistant H1299 cells (Fig. 6B and C). Beside BAX, we have also found that the expression of XIAP was inherently high in the untreated H1299 cells as shown in the heat map analysis (Fig. 6C). XIAP is a protein known for its role in inhibiting apoptosis through the blockade of caspases, and high expression of XIAP has been link to poor prognosis in majority of NSCLC samples [43, 44]. Interestingly, after initiation of curcumin and TRAIL, there was a significant decrease in the expression of XIAP in H1299 suggesting that the combined treatment were capable of sensitizing H1299 by lowering its anti-apoptotic activity for apoptosis to initiate [45, 46]. While these findings provide insight into transcriptional regulation of key apoptotic genes, further validation through protein-level analysis such as using western blot would strengthen the interpretation of these results and support the proposed mechanisms.

Kyoto Encyclopaedia of Genes and Genomes or KEGG pathway analysis was conducted to illustrate how different genes were involved in regulating apoptosis following the combined treatment of curcumin and TRAIL in TRAIL-resistance H1299 (Fig. 7). KEGG analysis in H1299 after TRAIL resulted in general reduction of apoptosis-related genes including the initiator and effector caspases suggesting the characteristic of TRAIL- resistant in H1299 (supplement 1) [32, 47]. While TRAIL alone showed limited effects, the combined treatment of curcumin and TRAIL produced the most significant pro-apoptotic shift as an increased in the expression of several pro-apoptotic genes, including *BAX*,* BAK* (BCL2 Antagonist/Killer 1), *BAD* (BCL2 Associated Agonist of Cell Death), *BID *(BH3 Interacting Domain Death Agonist) and Cytochrome C were noticed in H1299 cell line (Fig. 7). Besides that, we also found that curcumin reduced the expression of survival-associated genes such as *BCL-XL *(B-cell lymphoma-extra large) and *XIAP* (X-linked inhibitor of apoptosis) in H1299 suggesting that curcumin promotes apoptosis by modulating both death receptor and mitochondrial pathways, even in the absence of a functional P53 in H1299 (supplement 2) [48]. Furthermore, the increased expression of TNF-α and GADD45 in H1299 after the combined treatment further supports the associations of stress-response mechanisms that contribute to apoptosis through p53-independent signalling, as shown by other p53-null cancer models [49, 50]. To further support the involvement of mitochondrial-mediated apoptosis, functional assays such as mitochondrial membrane potential assessment and cytochrome c release assays would provide additional mechanistic validation of the observed molecular changes. In addition, future studies using p53-restored isogenic models or comparison with p53-proficient cell lines may be required to identify and confirm the role of p53 in the observed apoptotic response.

## Conclusion

TRAIL alone showed limited efficacy in inducing apoptosis in TRAIL-resistant cell lines, whereas the addition of curcumin was associated with enhanced reduction in cell viability and increased apoptotic response in both TRAIL-sensitive and TRAIL-resistant NSCLC cell lines. Although the H1299 cell line is p53-deficient, the combination treatment was associated with enrichment of intrinsic apoptosis-related genes, as indicated by KEGG pathway analysis. These findings suggest that curcumin may contribute to the enhancement of TRAIL-induced apoptotic responses in TRAIL-resistant NSCLC cells under in vitro conditions. However, as studies combining curcumin with TRAIL remain largely limited to cell-based assays, with relatively few investigations conducted in in vivo models, further studies are required to better understand their combined effects in a physiological context.

## Supplementary Information

Supplementary Material 1.

Supplementary Material 2.

## Data Availability

No datasets were generated or analysed during the current study.

## Abbreviations

ABL1: ABL proto-oncogene 1, non-receptor tyrosine kinase
AIFM1: Apoptosis inducing factor mitochondria associated 1
AKT1: AKT serine/threonine kinase 1
ANOVA: Analysis of Variance
ATCC: American Type Culture Collection
AV: Annexin V
BAK1: BCL2 antagonist/killer 1
BAX: BCL2 Associated X, Apoptosis Regulator
BCL-2: B-cell lymphoma 2
BCL-XL: B-cell lymphoma-extra large
BCL2: BCL2 apoptosis regulator
BCL10: BCL10 immune signaling adaptor
BCL2L1: BCL2 like 1
BCL2L2: BCL2 like 2
BIK: BCL2 interacting killer
BNIP3: BCL2 interacting protein 3
BNIP3L: BCL2 interacting protein 3 like
CASP9: Caspase 9
CIDEA: Cell death-inducing DNA fragmentation factor, alpha subunit-like effector A
cDNA: Complementary DNA
cFLIP: Cellular FLICE-like inhibitory protein
CO₂: Carbon dioxide
Ct: Cycle threshold
DcR1: Decoy receptor 1
DcR2: Decoy receptor 2
DISC: Death inducing signaling complex
DMEM: Dulbecco’s Modified Eagle Medium
DMSO: Dimethyl sulfoxide
DPBS: Dulbecco’s Phosphate Buffered Saline
DR4: Death receptor 4
DR5: Death receptor 5
EDTA: Ethylenediaminetetraacetic acid
FADD: Fas-associated protein with death domain
FBS: Fetal bovine serum
FGF: Fibroblast growth factor
GADD45: Growth arrest and DNA damage-inducible 45
GADD45A: Growth arrest and DNA damage inducible alpha
IC50: Inhibitory concentration 50
KEGG: Kyoto Encyclopedia of Genes and Genomes
KRAS: Kirsten rat sarcoma viral oncogene homolog
LTA: Lymphotoxin alpha
LTBR: Lymphotoxin beta receptor
MSC1: Myeloid cell leukemia 1
MSCs: Mesenchymal stem cells
MTS: 3-(4,5-dimethylthiazol-2-yl)-5-(3-carboxymethoxyphenyl)-2-(4-sulfophenyl)-2 H-tetrazolium
NSCLC: Non-small cell lung cancer
PBS: Phosphate-buffered saline
PCR: Polymerase chain reaction
PE: Phycoerythrin
PI: Propidium iodide
rhEGF: Recombinant epidermal growth factor
rhTRAIL: Recombinant human TRAIL
ROS: Reactive oxygen species
RPMI 1640: Roswell Park Memorial Institute Medium 1640
SSC: Side scatter
TNF: Tumor necrosis factor
TNF-α: Tumor necrosis factor alpha
TNFRSF1B: TNF receptor superfamily member 1B
TNFRSF10B: Tumor necrosis factor receptor superfamily, member 10b
TRAF2: TNF receptor associated factor 2
TRAF3: TNF receptor associated factor 3
TRAIL: Tumor necrosis factor-related apoptosis-inducing ligand
TP73: Tumor protein p73
XIAP: X-linked inhibitor of apoptosis
